# Electronic Cigarettes and Female Reproduction: A Systematic Review of Experimental and Clinical Evidence

**DOI:** 10.3390/ijms27146528

**Published:** 2026-07-22

**Authors:** Maria Cristina Budani, Gian Mario Tiboni

**Affiliations:** 1 School of Medicine and Health Sciences, University “G. d’Annunzio” Chieti-Pescara, Via dei Vestini 29, 66100 Chieti, Italy; 2 Department of Medical, Oral and Biotechnological Sciences, University “G. d’Annunzio” Chieti-Pescara, Via Dei Vestini 31, 66100 Chieti, Italy; tiboni@unich.it

**Keywords:** electronic cigarettes, female fertility, reproduction, assisted reproductive technology, in vitro fertilization, pregnancy, embryo development

## Abstract

Electronic cigarettes (e-cigarettes) have gained widespread diffusion as a perceived safer alternative to conventional smoking, particularly among women of reproductive age. The present systematic review provides an overview of the current literature on the effects of e-cigarette exposure on female reproductive function, embryonic development, and pregnancy outcomes, with a focus on underlying molecular mechanisms. A systematic literature search was conducted in PubMed, Web of Science, and Google Scholar, and the review was conducted in accordance with the PRISMA 2020 guidelines. A total of 11 studies met the eligibility criteria and were included in the qualitative synthesis, comprising six experimental and five clinical studies. Experimental studies suggest that e-cigarette exposure may impair ovarian function, leading to morphological alterations, disrupted folliculogenesis, and altered steroidogenesis, mainly through oxidative stress, apoptosis, mitochondrial dysfunction, and dysregulation of pathways such as Hippo signaling. Non-nicotine components, including propylene glycol (PG), vegetable glycerin (VG), and flavoring agents, appear to be key contributors. Adverse effects on implantation, embryo development, and placental growth have also been reported in experimental models, although further studies are needed. Clinical evidence remains scarce, suggesting possible associations between e-cigarette smoking and reduced ovarian reserve and fecundability in naturally conceiving women. No conclusive associations have been identified with in vitro fertilization outcomes. Overall, e-cigarette exposure may negatively affect female reproductive function, although the predominance of experimental data and the limited clinical evidence preclude definitive conclusions.

## 1. Introduction

Conventional cigarette smoking represents a serious public health concern and an established risk factor for cancer, cardiovascular disease, and respiratory disorders [1]. Although the global smoking prevalence decreased from 22.7% in 2007 to 17% in 2021, the total number of smokers remains high, with at least 940 million men and 193 million women aged 15 years and older in 2019 [1]. Cigarette smoke contains several toxic substances, including nicotine, polycyclic aromatic hydrocarbons (PAHs), tobacco-specific nitrosamines, carbon monoxide, aldehydes, and heavy metals, which also exert harmful effects on the female reproductive system [2].

The gonadotoxic effects of cigarette smoking have been extensively investigated. Several components of cigarette smoke, including nicotine, cotinine, cadmium, and benzo[a]pyrene, have been detected in follicular fluid, thereby directly exposing the oocytes to these harmful substances within their microenvironment [2,3]. Consistent with these findings, cigarette smoke and its constituents have been shown to interfere with key processes of ovarian function, including gametogenesis, steroidogenesis, and folliculogenesis [3], with close association between tobacco smoke and accelerated follicle loss, abnormal follicle growth and impairment of oocyte morphology and maturation [3].

From a clinical perspective, epidemiological data indicate that a cigarette smoking habit is associated with delayed conception of approximately 6–12 months, an earlier onset of menopause by about 3–4 years, and reduced fertility in both naturally conceiving women and those undergoing in vitro fertilization (IVF) techniques [4,5].

In recent years, the use of electronic cigarettes (e-cigarettes) has rapidly increased as a perceived less harmful alternative to conventional cigarettes. E-cigarettes generally contain fewer toxic compounds than traditional cigarettes, but several studies have shown that the aerosol produced may contain nicotine, aldehydes, heavy metals and other potentially harmful substances, with possible negative effects on the cardiovascular and respiratory systems [6,7].

Recently, interest in the effects of e-cigarettes on female reproductive health has increased, particularly given the rising prevalence of e-cigarette use among women of reproductive age. This is particularly concerning given the potential implications in female reproductive function and pregnancy outcomes, which remain poorly understood.

Given the emerging nature of this field, this review aims to summarize the available literature, with particular emphasis on the underlying molecular mechanisms.

## 2. Materials and Methods

### 2.1. Reporting Guideline and Data Items

The Preferred Reporting Items for Systematic Reviews and Meta-Analyses (PRISMA) 2020 guidelines were followed [8]. The PRISMA checklist is provided as Supplementary Material (S1). The present systematic review provides an overview of the experimental evidence on the effects of e-cigarette exposure on female reproductive health, including ovarian function, embryonic development, and pregnancy outcomes. In addition, clinical evidence regarding the potential impact of e-cigarette exposure on female fertility and IVF technique outcomes was evaluated.

### 2.2. Information Sources, Search Strategy

A comprehensive literature search was conducted using the PubMed, Web of Science, and Google Scholar databases. The search included articles published up to April 2026. The search terms for each database were as follows: “electronic cigarette”, “e-cigarette”, “vaping”, “female fertility”, “ovarian function”, “folliculogenesis”, “steroidogenesis”, “embryonic development”, “implantation”, “pregnancy outcomes”, “assisted reproductive technology”, and “in vitro fertilization”.

### 2.3. Eligibility Criteria

For the purpose of the present review, studies were considered eligible if they met the following inclusion criteria: original peer-reviewed research articles, experimental studies conducted in mammalian or non-mammalian models, observational or clinical studies conducted in humans, studies investigating the effects of e-cigarette exposure on female reproductive function, ovarian physiology, embryonic development, implantation, pregnancy outcomes or IVF outcomes.

Studies were excluded if they were review articles, systematic reviews, meta-analyses, editorials, letters, commentaries, or conference abstracts, non-peer-reviewed publications, studies primarily focused on male reproductive health, or studies investigating health outcomes unrelated to female reproduction. In addition, studies reporting exclusively neonatal or perinatal outcomes (birth weight, gestational age, or other obstetric endpoints) were not considered eligible.

### 2.4. Selection Process

All records were independently screened by two reviewers. After duplicate removal, titles and abstracts were evaluated according to the predefined eligibility criteria. Articles considered potentially relevant underwent full-text assessment for eligibility. Studies fulfilling the inclusion criteria were included in the study.

### 2.5. Data Collection Process

The complete record of each study was reviewed, and the following data were extracted: first author, year of publication, study design (experimental or clinical study), characteristics of e-cigarette exposure, reproductive outcomes, and main findings. The included studies were subsequently grouped according to the type of evidence (experimental or clinical) and synthesized descriptively.

### 2.6. Effect Measures

Effect measures were extracted as originally reported in the included studies. In experimental studies, extracted data included findings related to ovarian morphology, folliculogenesis, steroidogenesis, embryonic development, implantation, and placental growth, as well as molecular pathways involved in the biological effects of e-cigarette exposure. In clinical studies, extracted data included fecundability ratios, hormone levels [as well as anti-Müllerian hormone (AMH) and follicle-stimulating hormone (FSH) levels], oocyte yield, embryo development outcomes, implantation rates, pregnancy outcomes, and live birth rates.

### 2.7. Synthesis Methods

Studies were grouped according to design (experimental or clinical) and further categorized based on the main reproductive outcomes investigated (including ovarian function, embryonic development, and pregnancy outcomes). Within each category, findings were synthesized descriptively, with particular attention to the proposed molecular mechanisms underlying the effects of e-cigarette exposure on female reproductive health.

### 2.8. Quality Assessment

The risk of bias assessment was performed only for the clinical studies. For this purpose, the Newcastle–Ottawa Scale (NOS) instrument was used in order to assess the quality of studies [9]. This scale assigns a maximum of 9 points to each study: 4 for exposure selection and assessment, 2 for comparability, and 3 for outcome assessment. If a study receives a score of ≥6, it is considered a study with a low risk of bias. The quality assessment was performed independently by two researchers. Any discrepancies were resolved through discussion and consensus.

## 3. Results

### 3.1. Study Selection

The literature search identified a total of 141 records through electronic database searching. After removal of 82 duplicate records, 59 articles underwent title and abstract screening. Following the initial screening, 45 records were excluded because they did not meet the predefined eligibility criteria. The remaining 14 full-text articles were assessed for eligibility. At full-text assessment, three studies were excluded [10,11,12]. One study was a protocol with no available results [10], one reported exclusively neonatal/perinatal outcomes [11], and one evaluated only biomarkers of exposure without assessing reproductive endpoints [12].

**Figure 1:**
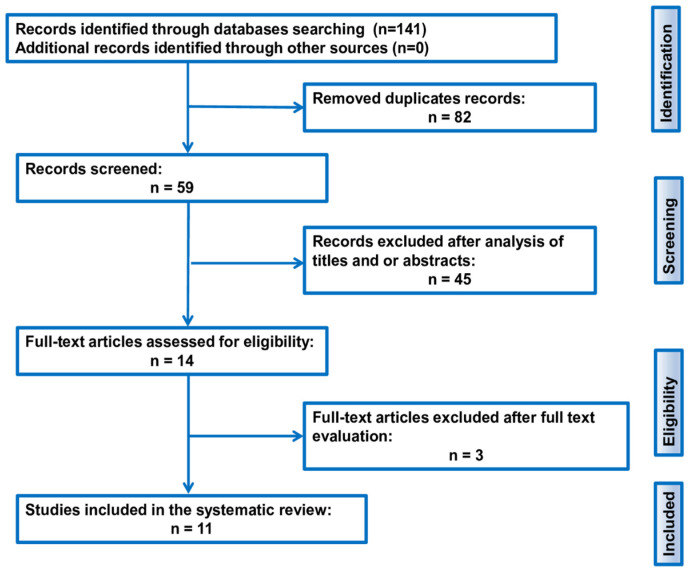
Flow chart of the study selection.

Ultimately, 11 studies met the inclusion criteria and were included in the study [13,14,15,16,17,18,19,20,21,22,23], comprising six experimental studies [13,14,15,16,17,18] and five clinical studies [19,20,21,22,23]. The study selection process is illustrated in the PRISMA 2020 flow diagram (Figure 1).

### 3.2. Characteristics of the Included Studies

A total of 11 studies fulfilled the eligibility criteria and were included in the study [13,14,15,16,17,18,19,20,21,22,23].

The experimental evidence included in vitro and in vivo studies conducted in mammalian [13,14,17,18] and non-mammalian models, such as chicken embryos [15] and Xenopus laevis [16]. Among mammalian models, most studies used murine or rat systems to investigate the effects of whole e-cigarette vapor exposure or specific e-liquid components on female reproductive function. These studies primarily focused on ovarian morphology, follicular development, and steroidogenesis [13,14,17,18]. Non-mammalian models were used to evaluate early embryonic development, vascular development, and craniofacial morphogenesis under exposure to e-cigarette aerosol or e-liquid-derived compounds [15,16].

Exposure conditions varied considerably across the experimental studies, ranging from whole e-cigarette aerosol exposure to complete e-liquids and isolated e-liquid constituents, including nicotine, propylene glycol (PG), vegetable glycerin (VG), and flavoring agents. The detailed characteristics of the studies are addressed in Table 1.

The clinical studies comprised prospective [19,21,22] and retrospective observational [20,23] studies evaluating women attempting natural conception [19,20] or undergoing IVF techniques [21,22,23]. One study also evaluated the potential impact of male partner e-cigarette use on IVF outcomes [23]. Table 2 summarizes the characteristics of the clinical studies included.

### 3.3. Quality Assessment of Clinical Studies

Overall, the clinical studies demonstrated moderate to high methodological quality, with NOS scores ranging from 7 to 9 out of 9. Two studies achieved the maximum score of nine stars [19,22], whereas the remaining studies scored between seven and eight stars, indicating an overall low risk of bias [20,21,23]. Differences in NOS scores were primarily related to study selection and population characteristics rather than to outcome assessment or comparability. Indeed, across all studies, comparability was generally adequate, as most studies adjusted for key confounding factors such as age, body mass index (BMI), and reproductive history. Outcome assessment was considered robust across all included studies, as it was based on clinically relevant reproductive outcomes. Detailed NOS scores for each study are presented in Table 3.

### 3.4. Synthesis of the Evidence

#### 3.4.1. Brief Composition of E-Cigarettes

E-cigarettes have evolved considerably in recent years, varying in design and functionality but maintaining basic functional elements: a cartridge with e-liquid, an atomizer, a sensor, and a battery [24]. First-generation devices were disposable, while later versions introduced refillable cartridges, replaceable batteries, and customizable e-liquids [6]. Briefly, upon activation, the battery powers the atomizer, which heats the e-liquid to generate an inhalable aerosol.

E-liquids generally contain water, glycols, and nicotine, although more than 80 compounds have been identified in both e-liquids and aerosol [25]. The main glycols, such as PG and VG, are present in varying concentrations and can degrade upon heating [6,7,25,26]. Nicotine content is highly variable, ranging from 0 to 35 mg per puff [6,25,27,28].

E-cigarette aerosol contains metals (lead, chromium, nickel) and various chemical compounds, including tobacco-specific nitrosamines, carbonyls, and volatile organic compounds such as benzene [6,7,29,30,31,32], although their presence and concentrations may vary substantially depending on device type and e-liquid composition [6,7,29,30,31,32]. Hydrocarbons and PAHs, including benzo[a]pyrene, as well as phenols, have also been detected [6,7,25,33].

#### 3.4.2. Impact of E-Cigarette Exposure on Ovarian Function: Experimental Data

##### E-Cigarette-Induced Ovarian Toxicity: Morphological Damages and Impaired Steroidogenesis

**Figure 2:**
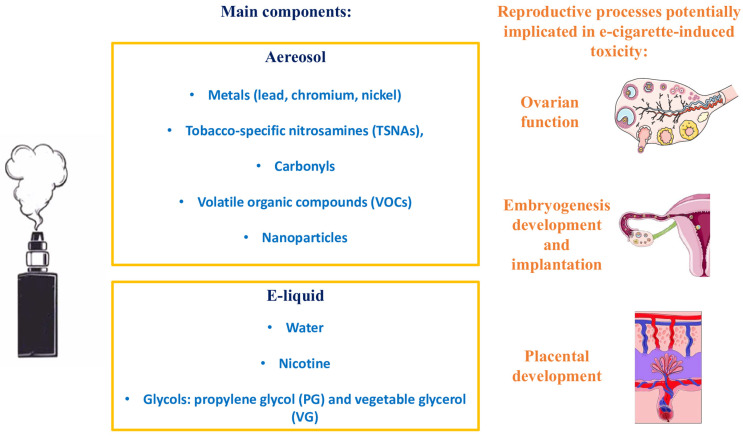
Overview of e-cigarette constituents and reproductive processes implicated in e-cigarette-induced toxicity. The figure illustrates the main constituents of e-cigarette aerosols, including nicotine, e-liquid solvents [propylene glycol (PG) and vegetable glycerin (VG)], flavoring agents, and aerosol-derived toxicants, such as metals and chemical by-products generated during heating. Ovarian function, embryonic development and implantation, and placental development represent the reproductive processes most commonly implicated in e-cigarette-induced toxicity. Image adapted from Servier Medical Art (https://smart.servier.com), licensed under CC BY 4.0 (https://creativecommons.org/licenses/by/4.0/) (accessed on 13 July 2026).

**Figure 3:**
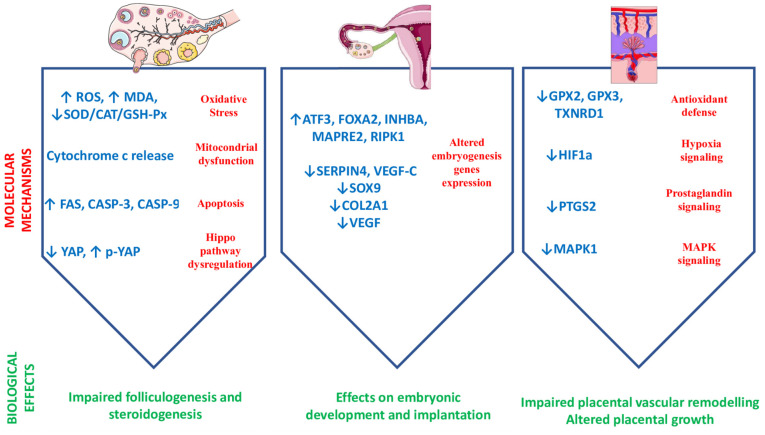
Molecular mechanisms and biological effects underlying e-cigarette–induced reproductive toxicity. Exposure to e-cigarette constituents may impair folliculogenesis, uterine receptivity, and placental growth through multiple molecular mechanisms. Experimental studies have implicated oxidative stress, mitochondrial dysfunction, and apoptosis together with dysregulation of signaling pathways (Hippo signaling) in ovarian damage and impaired follicular development. Altered implantation processes and placental growth have also been associated with dysregulated signaling pathways involved in uterine receptivity, vascular remodeling, hypoxia response, and oxidative stress regulation. Image adapted from Servier Medical Art (https://smart.servier.com), licensed under CC BY 4.0 (https://creativecommons.org/licenses/by/4.0/) (accessed on 13 July 2026).

The potential impact of e-cigarettes on female fertility is gaining increasing attention, despite scarce experimental evidence. The detailed description of the studies is summarized in Table 1. In addition, a schematic overview is provided in Figure 2, while Figure 3 presents a scheme of the alterations associated with e-cigarette exposure in the female reproductive system.

In the recent study by Chen et al. (2022) [13], ovaries from 21- and 35-day-old rats were divided into two exposure groups receiving 0.05 mg and 0.5 mg of e-liquids (containing 0.5 and 5 mg nicotine/kg, respectively). Dose-dependent morphological damage was highlighted after exposure. Specifically, granulosa cells (GCs) showed nuclear pyknosis, disorganization, and reduced cohesion at 0.05 mg exposure, while at 0.5 mg, more severe alterations were observed, including oocyte degeneration, extensive GC pyknosis, and theca layer abnormalities. Overall, both exposed groups exhibited fewer normal follicles and reduced estrogen production compared with controls [13].

Further investigation into individual e-liquid components indicates that nicotine alone exerts relatively limited ovarian toxicity, whereas other constituents, with particular attention to flavorings, PG, and VG, are major contributors to ovarian damage. Notably, low-nicotine (LN) e-liquids cause more severe ovarian damage than high-nicotine (HN) formulations, likely due to higher concentrations of non-nicotine components [14].

ATF3 (activating transcription factor 3), CASP3 (caspase-3), CASP9 (caspase-9), COL2A1 (collagen type II alpha 1 chain), FAS (Fas cell surface death receptor), FOXA2 (forkhead box A2), GPX2/GPX3 (glutathione peroxidase 2/3), GSH-Px (glutathione peroxidase), HIF-1α (hypoxia-inducible factor 1-alpha), INHBA (inhibin subunit beta A), MAPK1 (mitogen-activated protein kinase 1), MAPRE2 (microtubule-associated protein RP/EB family member 2), MDA (malondialdehyde), p-YAP (phosphorylated YAP), PTGS2 (prostaglandin-endoperoxide synthase 2), RIPK1 (receptor-interacting serine/threonine-protein kinase 1), ROS (reactive oxygen species), SERPIN4 (serpin family A member 4), SOD/CAT (superoxide dismutase/catalase), SOX9 (SRY-box transcription factor 9), TXNRD1 (thioredoxin reductase 1), VEGF (vascular endothelial growth factor), VEGF-C (vascular endothelial growth factor C), and YAP (Yes-associated protein).

##### E-Cigarette-Induced Ovarian Toxicity: Molecular Mechanisms

Among the molecular mechanisms potentially involved in ovarian toxicity induced by e-liquids, the dysregulation of the Hippo signaling pathway appears to play a central role [34].

The Hippo signaling pathway is a key regulator of follicular development [35], and its key mediators have been shown to be expressed in follicles at different developmental stages [36]. The Yes-associated protein (YAP) is a downstream effector of the Hippo signaling pathway and plays a pivotal role in ovarian development [36].

The study by Chen et al. (2022) [13] demonstrated that YAP expression in the ovaries of 21- and 35-day-old rats was significantly reduced following e-liquid exposure, particularly in the 0.5 mg group, while its phosphorylated form increased in a statistically significant manner. These findings suggest that e-liquids may exert a negative role on follicular growth [13].

Additionally, Chen et al. (2023) [14] highlighted a substantial contribution of non-nicotine components to ovarian damage. Exposure to flavorings, PG, and VG disrupted the oxidative balance in ovarian tissue by increasing reactive oxygen species levels and reducing antioxidant capacity. Additionally, LN e-liquids induced a more marked reduction in antioxidant defenses, including superoxide dismutase (SOD), catalase (CAT), and glutathione peroxidase (GSH-Px), as well as a stronger increase in lipid peroxidation, measured by malondialdehyde (MDA), compared with HN formulations. This oxidative imbalance was associated with increased expression of apoptosis markers, as evidenced by elevated Fas (Fas cell surface death receptor), cytochrome c (Cyc-c), caspase-3 (CASP3), and caspase-9 (CASP9), with the VG group showing the most pronounced effect [14].

#### 3.4.3. Impact of E-Cigarette Exposure on Embryonic Development in Non-Mammalian Models: Experimental Data

The process of embryogenesis has also been studied to determine whether e-cigarette exposure may influence developmental processes in experimental models. An overview of the included studies is provided in Table 1.

Ashour et al. (2020) [15] investigated the effects of direct e-liquid exposure in chicken embryos treated at 3 days of incubation and monitored for 5 days, reporting a dose-dependent embryonic mortality. Specifically, 76% of embryos treated with 60 µg e-liquid died, compared to 64% in the 30 µg e-liquid group and 9% in the control group [15].

At the molecular level, key regulatory genes of embryogenesis were altered, with activating transcription factor 3 (ATF-3), forkhead box A2 (FOXA2), inhibin subunit beta A (INHBA), microtubule-associated protein RP/EB family member 2 (MAPRE-2), and receptor-interacting serine/threonine-protein kinase 1 (RIPK-1) being upregulated, while serpin family E member 4 (SERPIN-4) and vascular endothelial growth factor C (VEGF-C) were downregulated in embryos treated with e-liquid compared with their matched controls [15]. The process of angiogenesis in the chorioallantoic membrane (CAM) was also impaired, with fewer junctions, shorter vessel length, and reduced vessel area at high concentrations [15].

In the Xenopus laevis model, exposure to e-cigarette aerosol mixtures (e-cigAMs) has been associated with craniofacial and developmental defects, including alterations in cartilage, muscle, and vascular structures [16]. These mixtures varied in nicotine concentration, PG/VG ratios, and flavor composition, ranging from simple formulations to complex blends containing multiple flavoring compounds. Nicotine/PG/VG-only mixtures induced mild, dose-dependent craniofacial alterations, mainly affecting mouth morphology and midface width. In contrast, flavored and chemically complex mixtures produced more severe phenotypes, including midface hypoplasia, altered mouth shape, and median clefts, even in the absence of nicotine. These effects were accompanied by disrupted craniofacial tissue organization and impaired neural crest cell function, with downregulation of key genes involved in cartilage development, including SRY-box transcription factor 9 (SOX9) and collagen type II alpha 1 chain (COL2A1), as well as vascular development, including vascular endothelial growth factor (VEGF) [16].

#### 3.4.4. In Vivo Exposure to E-Cigarettes in Mammalian Models

##### In Vivo Exposure to E-Cigarettes: Effects on Reproduction, Implantation, and Pregnancy Outcomes

Emerging data from in vivo studies using mammalian models suggested that e-cigarette exposure can adversely affect reproductive function, implantation success, and pregnancy outcomes. A detailed summary of the studies is provided in Table 1.

Wetendorf et al. (2019) [17] used a murine model in which C57BL/6J females were exposed to e-cigarette vapor (PG/VG 55:45, 24 mg/mL nicotine, 3 h/day, 5 days/week). Females were exposed from the first day of mating for a total of four months, resulting in a significant delay in the onset of the first litter, suggestive of reduced fecundity. More to the point, in implantation studies, mice were pre-exposed for four weeks prior to mating and continuously exposed during early pregnancy. E-cigarette-exposed mice exhibited a delay in embryo attachment, as few embryos implanted by day 5.5 of pregnancy. Long-term effects included reduced body weight in female offspring [17].

Recently, in the study by Marbrey et al. (2025) [18], female C57BL/6J mice were exposed to flavored e-cigarette vapor, with (6 mg/mL) or without nicotine (3 h per day, 5 days per week) beginning three weeks prior to mating and continuing during pregnancy. Exposure resulted in significantly elevated serum cotinine levels in nicotine-treated animals, confirming systemic uptake, but did not affect litter size. At early gestational stage day 6.5, nicotine-free vapor exposure was associated with increased erythrocyte accumulation at implantation sites, suggesting impaired vascular remodeling. At day 12.5 of gestation, no significant differences in overall embryo or placental weights were observed; however, embryo-to-placental weight ratios were reduced in nicotine-exposed groups [18].

##### In Vivo Exposure to E-Cigarettes: Molecular Alterations Affecting the Processes of Embryo Implantation and Placental Growth

E-cigarette exposure may interfere with the processes of embryo implantation and placental growth, as illustrated in Figure 3.

To assess its effects on implantation, uteri from e-cigarette–exposed mice at pseudopregnant day 4.5 were analyzed. Exposure was associated with dysregulation of genes involved in uterine receptivity, including those related to integrin signaling, prostanoid biosynthesis, cell proliferation, JAK signaling, and chemokine activity. In addition, the tight junction protein claudin-10 (CLDN10) was upregulated in both epithelial and stromal compartments, with increased mRNA expression, suggesting potential uterine remodeling following e-cigarette exposure [17].

Extending these observations to later stages of pregnancy, Marbrey et al. (2025) [18] reported that e-cigarette exposure reduced the expression of genes involved in hypoxia response, oxidative stress regulation, and placental growth in placental tissue at gestational day 12.5, with effects varying by fetal sex. Key downregulated genes included hypoxia-inducible factor 1 alpha (HIF1A), prostaglandin-endoperoxide synthase 2 (PTGS2), glutathione peroxidases 2 and 3 (GPX2/3), thioredoxin reductase 1 (TXNRD1), and mitogen-activated protein kinase 1 (MAPK1) [18].

#### 3.4.5. Impact of E-Cigarette Exposure on Female Fertility and In Vitro Fertilization (IVF) Outcomes: Clinical Evidence

The current clinical evidence on the effects of e-cigarette use on female fertility and reproductive outcomes is still limited and inconsistent. The available studies are summarized in Table 2.

Among women attempting a natural conception, the prospective cohort study PRESTO included 4586 women aged 21–45 years in the United States and Canada. E-cigarette use (never, former, current) was assessed over 12 months, and fecundability ratios (FRs) were estimated based on time to pregnancy. E-cigarette use was associated with a non-statistically significant trend toward a reduction in fecundability [19]. Consistently, a large retrospective study of 21,102 women in the United Kingdom reported reduced ovarian reserve among both smokers and e-cigarette users, as indicated by lower AMH levels, although no effect was observed in terms of FSH levels [20].

Moving from naturally conceiving women to those undergoing IVF techniques, the available evidence remains scarce and heterogeneous across studies. Notably, nicotine and cotinine were detected in the follicular fluid of e-cigarette users, albeit at lower concentrations than in conventional smokers [21]. However, no significant differences in oocyte yield, embryo development, or IVF outcomes were observed among e-cigarette users [21,22]. Only the number of germinal vesicle (GV) oocytes appeared statistically higher in e-cigarette smokers compared to conventional cigarette users [22]. Finally, considering the potential contribution of male partners, a retrospective study comparing IVF outcomes based on male partners’ use of e-cigarettes versus conventional cigarettes found that e-cigarette users had higher sperm motility, lower prolactin levels, lower miscarriage and higher live birth rates compared to conventional smokers, with no major differences in fertilization parameters [23].

## 4. Discussion

The current body of evidence on e-cigarette exposure and its effects on female reproductive health and pregnancy outcomes suggests a potential for adverse biological effects. However, conclusions remain limited due to the predominance of experimental data, methodological discrepancies between studies, and the scarcity of robust clinical data.

Emerging evidence highlights the sensitivity of ovarian tissue to e-liquid exposure in vitro [13,14]. Dose-dependent alterations in follicular architecture, reduced granulosa cell integrity, and impaired steroidogenesis suggest that ovarian function may be affected not only by nicotine but also by other constituents of e-cigarettes. These findings point to a potential role of PG, VG, and flavoring agents, although their specific contributions to ovarian toxicity remain to be fully elucidated.

In addition to evidence suggesting potential ovarian toxicity, studies across different experimental models indicate that e-cigarette exposure may also exert detrimental effects on pregnancy, ranging from impaired uterine receptivity and defective implantation to abnormal embryonic development and altered placental growth [15,16,17,18].

The biologically plausible mechanisms underlying these effects involve a shared network of pathways that appear to be modulated by e-cigarette exposure, with consequences for both ovarian and uterine functions [13,14,17,18]. In particular, altered oxidative stress homeostasis, increased apoptosis, and mitochondrial dysfunction represent central events contributing to tissue damage across reproductive compartments. In the ovary, these mechanisms may impair follicular development, steroidogenesis, and oocyte quality [13,14], while in the uterus and placenta, they may disrupt endometrial receptivity, implantation processes, and vascular remodeling [17,18].

Despite these preliminary findings, the current evidence is characterized by marked heterogeneity across studies in terms of experimental models, exposure conditions, and e-liquid composition, which limits definitive risk assessment and hinders a clear definition of the underlying molecular mechanisms. From a methodological perspective, the experimental studies provided important mechanistic insights under controlled exposure conditions, representing a major strength of the available evidence. However, differences in animal models, exposure protocols, and e-liquid formulations substantially limited the comparability of findings and their translation to human reproductive physiology. An additional limitation of the available literature is the lack of standardization in exposure doses and reporting of e-cigarette constituents across studies. Concentrations of nicotine, PG, VG, and flavoring agents vary widely depending on device type, usage patterns, and experimental conditions, making direct comparisons between studies difficult.

Additionally, real-world exposure involves a complex mixture of chemical compounds. The potential interactions among the toxic substances of e-cigarettes, including additive or synergistic toxic effects, remain poorly understood in the context of female reproductive health.

Nevertheless, the available data collectively support caution and highlight e-cigarettes as a potential reproductive toxicant requiring further rigorous investigation. More to the point, while experimental studies consistently suggest potential adverse effects of e-cigarette exposure on female reproduction, these findings cannot be directly extrapolated to humans. Clinical evidence remains limited, heterogeneous, and largely inconclusive, thereby precluding definitive conclusions.

Observational studies in naturally conceiving women have suggested potential associations between e-cigarette use, reduced AMH levels, and decreased fecundability, as reflected by longer time to pregnancy [19,20]. Notably, no consistent associations have been reported with IVF outcomes, although one study based on the type of smoking reported by the male partners (conventional cigarette versus e-cigarette use) showed a lower clinical miscarriage and a higher live birth rate in the e-cigarette group during the IVF cycle [23]. While these findings may suggest a different reproductive risk profile among e-cigarette users compared with conventional smokers, they should not be interpreted as evidence of a beneficial effect of e-cigarette use. Rather, they should be considered preliminary observations that require further investigation.

It is noteworthy that, although the clinical studies generally showed a low risk of bias according to the Newcastle–Ottawa Scale, the available evidence remains limited by the small number of studies, heterogeneous populations, and reliance on self-reported e-cigarette use. Indeed, the limited amount of data, together with substantial heterogeneity in vaping behaviors among women, should be considered key factors contributing to the preliminary nature of current conclusions. Furthermore, self-reported measures are unable to capture differences in vaping frequency, device characteristics, e-liquid composition, and actual nicotine exposure, potentially introducing substantial exposure misclassification and limiting the interpretation of clinical findings. Notably, the investigation of Traphoff et al. (2024) [21] represents, to date, the only study to directly quantify nicotine and cotinine levels in follicular fluid. Although not significantly different, nicotine and cotinine concentrations in follicular fluid were three- to six-fold higher in the e-cigarette group compared with non-smoking patients [21]. This underscores the need for future studies to incorporate biochemical measurements in biological fluids to more accurately evaluate the reproductive effects of e-cigarette use and strengthen causal implication. Another important aspect in the interpretation of the available evidence is that, in real-world settings, e-cigarette use is often not exclusive. Dual use of e-cigarettes and conventional cigarettes represents a common pattern of exposure, with potential sequential or concurrent exposure to multiple toxicants. This aspect should be considered when interpreting the translational relevance of the current evidence base.

## 5. Conclusions

In conclusion, despite the limited studies available, current findings highlight e-cigarettes as a potential reproductive hazard that warrants urgent investigation. In particular, the emerging role of non-nicotine components as significant drivers of reproductive toxicity underscores the importance of investigating the complex chemical composition of e-cigarettes, with specific attention to their contribution to reproductive impairment. Collectively, these data strongly support the need for further well-designed mechanistic and clinical studies, as the potential reproductive risks associated with e-cigarette use cannot be disregarded. Additionally, future research should prioritize the incorporation of biomarker-based exposure assessment to improve exposure characterization, and the development of standardized experimental exposure models to enhance comparability across studies.

## Figures and Tables

**Table 1:** Experimental studies investigating the effects of e-cigarettes on female fertility, embryo development and pregnancy outcomes.

Authors	Model	Animals	E-Liquids	Exposure	Results
Chen et al., 2022 [13]	In vitro	Rats: *n* = 36 21-day-old female rats (prepubertal) *n* = 36 35-day-old female rats (puberty)	E-liquids: nicotine salts (1.8%), VG (40%), PG (50%), distilled water (5%), and flavoring agents (3.2%)	Ovaries from 21- and 35-day-old rats divided into three groups: - 0.05 mg (e-liquids containing 0.5 of nicotine/kg) - 0.5 mg (e-liquids containing 5 mg of nicotine/kg) - control (no intervention)	- 0.05 mg exposure: increased GC pyknosis, disorganization, and reduced cell cohesion - 0.5 mg exposure: more severe damage, including oocyte degeneration, extensive GC pyknosis, and abnormal theca layer - Percentage of normal follicles decreased in both exposure groups (dose-dependent) - YAP expression was reduced in both exposure groups compared to controls - CYP19 expression was reduced in both exposure groups compared to controls - Protein analysis showed decreased CYP19, LATS2, and YAP, with increased phosphorylated forms (p-LATS2 and p-YAP) - Estradiol levels were decreased in both exposure groups compared to controls
Chen et al., 2023 [14]	In vitro	Rats: *n* = 189 35-day-old female rats	For 1 mL LN e-liquid: nicotine salt (10 mg dissolved in PG), VG (40%, 400 μL), PG (50%, 500 μL), distilled water (5%, 50 μL), and tobacco flavoring agent (5%, 50 μL) For 1 mL HN e-liquid: nicotine salt (50 mg dissolved in PG), VG (40%, 400 μL), PG (50%, 500 μL), distilled water (5%, 50 μL), and tobacco flavoring agent (5%, 50 μL).	Ovaries were divided into seven groups for intervention culture: Nicotine groups: doses of 0.5 mg/kg, and 0.05 mg/mL of nicotine VG, PG, and flavoring groups: 2 μL of VG/mL, 2.5 μL of PG/mL, and 0.25 μL of flavoring/mL added to the medium. LN e-liquid group: 0.05 mg/mL of nicotine, 0.25 μL of flavoring/mL, 2.5 μL of PG/mL, 2 μL of VG/mL, and 0.25 μL of distilled water/mL of medium. HN e-liquid group: 0.05 mg/mL of nicotine, 0.05 μL of flavoring/mL, 0.5 μL of PG/mL, 0.4 μL of VG/mL, and 0.05 μL of distilled water/mL of medium Control group: no intervention	- Nicotine group: morphology comparable to control, with preserved follicular structure, normal GC arrangement, minimal pyknosis, and intact theca layer closely associated with GCs - Flavoring group: abnormal follicular morphology with disruption of both GC and theca layers, showing structural irregularities and partial detachment between layers - PG group: altered morphology with extensive abnormalities in the GC layer, evident GC loss, and partial separation of the theca layer from GCs - VG group: severe structural damage with irregular follicle shape, highly disorganized and scattered GCs, extensive GC loss, and marked theca abnormalities including cell loss and widespread detachment from the GC layer - LN e-liquid group: pronounced damage characterized by large areas of disorganized GC layer, extensive GC loss, and severely altered theca layer with significant cell loss and clear separation from GCs - HN e-liquid group: moderate alterations with partially abnormal GC layers but overall more preserved organization compared to LN group, less pronounced GC loss, and milder theca disruption with limited separation - Nicotine group showed no significant changes in antioxidant enzymes (SOD, CAT, GSH-Px); in contrast, flavoring, PG, VG, and LN groups showed reduced antioxidant enzyme levels and increased MDA; HN group also showed decreased antioxidant defenses and increased MDA, but to a lesser extent than LN - Nicotine group showed minimal changes in apoptotic markers, with only Fas increased; flavoring, PG, VG, and LN groups exhibited progressive increases in apoptotic markers (Cyc-c, CASP3, CASP9, Fas); HN group also showed elevated apoptosis markers, though less pronounced than LN group
Ashour et al., 2020 [15]	In vivo	Chicken: *n* = 25 chicken embryos were treated at day 5 of incubation and observed for 48 h before being examined for the effect of ECL on angiogenesis *n* = 72 chicken embryos were exposed at day 3 of incubation and observed for another 5 days for survival analysis	E-cigarette liquid (ECL): nicotine concentration of 6 mg/mL in 70%, 30%, and 15% of propylene glycol (organic compound), vegetable glycerin, and fruit flavor, respectively.	Embryos treated with 30 or 60 µg suspension of final nicotine concentration from ECL: *n* = 30 embryos treated with 60 µg, *n* = 31 embryos treated with 30 µg *n* = 11 controls	- Dose-dependent increase in embryonic mortality: 64% (30 µg), 76% (60 µg), 9% (control); most deaths within 24 h; significantly reduced survival - No major morphological alterations observed in surviving embryos (heart, brain, liver) - Consistent gene expression changes essential in the embryogenesis process, with increased ATF-3, FOXA2, INHBA, MAPRE-2, and RIPK1, and decreased SERPINA-4 and VEGF-C - Impaired angiogenesis in CAM, with reduced vessel junctions and total vessel length, and decreased vessel area at higher concentration
Kennedy et al., 2018 [16]	In vivo	*Xenopus leavis*	Nicotine-containing and nicotine-free formulations composed of PG and VG in varying ratios, with or without flavor additives (tobacco, fruit, menthol, dessert-like blends)	Embryos exposed to e-cigAM at developmental stages from 2 cells (1.5 hpf) to stage 43 (87 hpf) or from stage 22–24 (26 hpf) until stages 43–45 (88–98 hpf).	- Craniofacial defects associated with e-cigarette aerosol exposure, accompanied by cranial cartilage and muscle abnormalities and reduced facial vascularization - Similar craniofacial phenotypes across different e-cigarette aerosol mixtures, with subtle variations related to e-liquid composition - Reduced expression of vascular and cartilage differentiation markers in neural crest cell line - Nicotine alone did not induce craniofacial defects but potentiated the effects of other e-liquid components
Wetendorf et al., 2019 [17]	In vivo	Mouse (C57BL/6J)	PG/VG mixture was in a 55:45 ratio with 24 mg/mL nicotine. No flavorings or other adulterants added	Whole-body inhalation for 3 h/d with two puffs per minute. Puff duration was 2 s. For fertility study: females were mated and exposed on the first day of mating to e-cigarette or sham for 5 days a week, for 4 months. For implantation study: females were primed with e-cigarettes 5 days a week, for 4 weeks, before mating to intact or vasectomized male	- Fertility outcomes: Slight decrease in pups per litter (not significant). No change in pup weight. Significant delay in first litter onset (3–4 days) - Implantation/early pregnancy: At day 5.5, almost complete absence of implantation sites in exposed mice. Normal progesterone levels confirmed pregnancy. Impaired embryo attachment. - Uterine molecular changes: Altered pathways include integrin, prostanoid biosynthesis, proliferation, JAK and chemokine signaling. Increased CLDN10 expression in epithelium and stroma. Altered uterine receptivity. - In utero exposure (F2 female fertility): No significant effects on reproductive outcomes. Female fertility preserved. - In utero exposure (F2 male fertility): Slight reduction in offspring number and weight (not significant). Delayed first litter in some cases. No changes in sperm parameters or testis morphology.
Marbrey et al., 2025 [18]	In vivo	Mouse (C57BL/6J)	Commercial flavored e-liquids (strawberry custard), with/without nicotine (0–6 mg/mL)	Whole-body exposure to flavored e-cigarette vapor (with or without nicotine, 6 mg/mL), 3 h/day, 5 days/week for ≥3 weeks before mating and continued during pregnancy (up to gestational day 6.5 or 12.5)	- E-cigarette exposure with nicotine significantly increased serum cotinine levels, while no differences were observed between nicotine-free and control groups - Litter size was not affected by exposure - Nicotine-free exposure was associated with increased erythrocyte accumulation at implantation sites, whereas nicotine exposure showed reduced accumulation compared to controls - Embryo elongation showed reduced variability and increased homogeneity in exposed groups, with a more pronounced effect in the nicotine group - At gestational day 12.5, no significant differences were observed in embryo or placental weights, but the embryo-to-placenta weight ratio was reduced in the nicotine group at the fetal–placental unit level - No significant differences in fetal resorptions were detected - Placental structure (decidua, labyrinth, junctional zone) was not significantly altered - Placental gene expression was downregulated for HIF1A, PTGS2 (particularly in females), GPX2/GPX3, TXNRD1, and MAPK1, with some effects depending on sex and type of exposure

ATF3: Activating transcription factor 3; CAM: Chorioallantoic membrane; CASP3: Caspase 3; CASP9: Caspase 9; CAT: Catalase; CLDN10: Claudin 10; Cyc-c: Cytochrome c; CYP19A1: Cytochrome P450 family 19 subfamily A member 1; e-cigAM: e-cigarette aerosol mixture; Fas: Fas cell surface death receptor; FOXA2: Forkhead box A2; GC: granulosa cells; GPX2: Glutathione peroxidase 2; GPX3: Glutathione peroxidase 3; GSH-Px: Glutathione peroxidase; HIF1A: Hypoxia-inducible factor 1 subunit alpha; HN: high nicotine concentration; hpf: hours from fertilization; INHBA: Inhibin subunit beta A; LATS2: Large tumor suppressor kinase 2; LN: low nicotine concentration; MAPK1: Mitogen-activated protein kinase 1; MAPRE2: Microtubule-associated protein RP/EB family member 2; MDA: Malondialdehyde; PG: propylen glycole; PTGS2: Prostaglandin-endoperoxide synthase 2; RIPK1: Receptor-interacting serine/threonine kinase 1; SERPINA4: Serpin family A member 4; SOD: Superoxide dismutase; TXNRD1: Thioredoxin reductase 1; VEGF-C: Vascular endothelial growth factor C; VG: vegetable glycerin; YAP: Yes-associated protein.

**Table 2:** Clinical studies investigating the effects of e-cigarettes.

Authors	Study Design	Study Group	Exposure Assessment	Results
Harlow et al., 2020 [19]	Web-based prospective preconception cohort study (PRESTO)	*n* = 4586 women actively trying to conceive not using fertility treatment or contraception	Questionnaire assessing e-cigarette use in the previous 4 weeks Exposure categorized as ever/never, current/former/never, and by vaping intensity (<3 mL/day vs. ≥3 mL/day) No specific e-liquid composition reported	- E-cigarette use was associated with a reduction in fecundability, without evidence of a clear dose–response relationship - No consistent association was observed with vaping intensity or time-varying exposure - Combined use of e-cigarettes and combustible cigarettes showed no clear association with fecundability
Wainwright et al., 2024 [20]	Retrospective observational cohort study	*n* = 21,102 women	Questionnaire assessing vaping and smoking status Exposure categorized as: never, former (quit), occasional, and current use No specific e-liquid composition reported	- Vaping (current and occasional) and smoking were associated with significantly reduced AMH levels - Vaping showed no significant association with FSH levels, whereas smoking was associated with a modest increase in FSH values
Trapphoff et al., 2024 [21]	Prospective cohort study	*n* = 320 women undergoing IVF treatment due to male-factor infertility	Questionnaire assessing smoking status Exposure categorized as: non-smokers, exclusive e-cigarette and/or hookah users, conventional cigarette smokers (with or without additional vaping/hookah use) Nicotine and cotinine measured in follicular fluid using GC–MS	- No significant differences in IVF outcomes (oocyte yield, fertilization, embryo transfer, and clinical pregnancy rates) across exposure groups - Nicotine and cotinine were detected in follicular fluid of all groups, with highest levels in smokers and lower levels in exclusive e-cigarette users - No association was observed between follicular fluid nicotine/cotinine concentrations and reproductive or clinical outcomes - Benzo[a]pyrene undetectable in all samples
Galanti et al., 2023 [22]	Prospective observational study	*n* = 410 infertile women with idiopathic or tubal infertility	Questionnaire assessing smoking status Exposure classified as non-smokers vs. smokers, with smokers defined as ≥10 cigarettes/day or ≥10 e-cigarette/HnB products/day for ≥1 year No specific e-liquid composition reported	- IVF outcomes (e-cigarette vs. other smokers): e-cigarette users showed no significant differences in number of oocytes retrieved, MII oocytes, or fertilization rate compared with cigarette or heated tobacco users - E-cigarette users had a higher number of GV compared to cigarette smokers
Kim et al., 2025 [23]	Retrospective cohort study	*n* = 296 couples underwent IVF treatment with frozen embryo transfer cycles: Exposure groups based on male partner smoking status: *n* = 151: conventional cigarette smokers *n* = 145: electronic cigarette	Questionnaire assessing smoking status Exposure defined as exclusive use of either conventional cigarettes or e-cigarettes for at least 6 consecutive months prior to semen analysis and IVF procedures	- Lower sperm concentration and higher sperm motility in e-cigarette users vs. conventional smokers - No differences in biochemical, clinical, ongoing pregnancy or biochemical miscarriage rates - Lower clinical miscarriage rate in e-cigarette group (11.96% vs. 36.27%) - Higher live birth rate in e-cigarette group (55.86% vs. 41.06%)

AMH: Anti-Müllerian Hormone; FSH: Follicle-Stimulating Hormone; GC–MS: Gas Chromatography–Mass Spectrometry; GV: Germinal Vesicles; HnB: Heat-not-burn products; IVF: In Vitro Fertilization; MII: Metaphase II oocytes.

**Table 3:** Quality of the clinical studies (New Castle-Ottawa scale).

Study	Selection				Comparability	Outcome			Score Risk of Bias
	Representativeness of the Exposed Cohort	Selection of the Non-Exposed Cohort	Ascertainment of Exposure	Demonstration That Outcome of Interest Was Not Present at Start of Study	Comparability of Cohorts on the Basis of the Design or Analysis	Assessment of Outcome	Was Follow-Up Long Enough for Outcomes to Occur	Adequacy of Follow-Up of Cohorts	
Harlow et al. (2020) [19]	*	*	*	*	**	*	*	*	9 stars Low risk of bias
Wainwright et al. (2024) [20]		*	*	*	**	*	*	*	8 stars Low risk of bias
Trapphoff et al. (2024) [21]		*	*	*	*	*	*	*	7 stars Low risk of bias
Galanti et al. (2023) [22]	*	*	*	*	**	*	*	*	9 stars Low risk of bias
Kim et al. (2025) [23]		*	*	*	**	*	*	*	8 stars Low risk of bias

The methodological quality of the clinical studies was evaluated using the Newcastle–Ottawa Scale (NOS), which assigns up to nine stars across three domains: selection (maximum 4 stars), comparability (maximum 2 stars), and outcome (maximum 3 stars). A NOS score ≥ 6 stars indicates high methodological quality and a low risk of bias.

## Data Availability

No new data were created or analyzed in this study. Data sharing is not applicable to this article.

## Supplementary Materials

The following supporting information can be downloaded at: https://www.mdpi.com/article/10.3390/ijms27146528/s1.
